# Modification of Ammonia Decomposition Activity of Ruthenium Nanoparticles by N-Doping of CNT Supports

**DOI:** 10.1007/s11244-017-0806-0

**Published:** 2017-06-29

**Authors:** Tamsin E. Bell, Guowu Zhan, Kejun Wu, Hua Chun Zeng, Laura Torrente-Murciano

**Affiliations:** 1 Department of Chemical Engineering and Biotechnology, University of Cambridge, Philippa Fawcett Drive, Cambridge, CB3 0AS UK; 2 Department of Chemical and Biomolecular Engineering, National University of Singapore, 4 Engineering Drive 4, Singapore, 117585 Singapore

**Keywords:** Ammonia decomposition, Nitrogen-doped CNT, Ruthenium, In situ H_2_ production, Nitrogen, N-CNT

## Abstract

The use of ammonia as a hydrogen vector has the potential to unlock the hydrogen economy. In this context, this paper presents novel insights into improving the ammonia decomposition activity of ruthenium nanoparticles supported on carbon nanotubes (CNT) by nitrogen doping. Our results can be applied to develop more active systems capable of delivering hydrogen on demand, with a view to move towards the low temperature target of less than 150 °C. Herein we demonstrate that nitrogen doping of the CNT support enhances the activity of ruthenium nanoparticles for the low temperature ammonia decomposition with turnover frequency numbers at 400 °C of 6200 LH_2_ mol_Ru_^−1^ h^−1^, higher than the corresponding value of unmodified CNT supports under the same conditions (4400 LH_2_ mol_Ru_^−1^ h^− 1^), despite presenting similar ruthenium particle sizes. However, when the nitrogen doping process is carried out with cetyltrimethylammonium bromide (CTAB) to enhance the dispersion of CNTs, the catalyst becomes virtually inactive despite the small ruthenium particle size, likely due to interference of CTAB, weakening the metal–support interaction. Our results demonstrate that the low temperature ammonia decomposition activity of ruthenium can be enhanced by nitrogen doping of the CNT support due to simultaneously increasing the support’s conductivity and basicity, electronically modifying the ruthenium active sites and promoting a strong metal–support interaction.

## Introduction

The high energy density of hydrogen makes it an attractive alternative energy vector to produce CO_x_-free energy in a proton exchange membrane (PEM) fuel cell [1]. However, the uptake of hydrogen related technologies is being limited due to the reluctance of the public to accept the widespread use of a flammable gas, with particular concerns for its safe storage and transportation. Further to the safety issues, many hydrogen storage techniques fall short of the US Department of Energy 2015 target of 9 wt.% hydrogen [2]. Some existing physical storage technologies such as pressurised cylinders and adsorbent materials have exceeded this target but are unable to release the stored hydrogen at a sufficiently fast rate or require impractical conditions such as cryogenic temperatures [3]. An alternative method for hydrogen storage is chemically in hydrogen rich molecules, which are used in conjunction with catalysts to decompose the molecule into hydrogen on demand. Out of these molecules, ammonia is an attractive option as it has a high hydrogen content both volumetrically (17.6 wt.%) and gravimetrically (81 g L^−1^), a narrow flammability range compared to hydrogen and a well-established, existing transportation network [4–6]. Whilst ammonia represents a promising solution for hydrogen storage, many of the essential, associated technologies in the fields of catalysts, membranes, reactors and sustainable ammonia production are relatively under developed and urgently require significant advances. Herein, we present work focussed on the aspect of development of catalysts for ammonia decomposition for in-situ hydrogen production at low temperatures.

In order for ammonia to be a viable vector for hydrogen storage, the decomposition temperature needs to be aligned with the operation temperature of the PEM fuel cell of  90–180 °C [7]. However, since ammonia decomposition is thermodynamically limited at low temperatures the majority of studies focus on high temperature ammonia decomposition [6, 8]. To date, the most active catalysts for ammonia decomposition are based on ruthenium supported on carbon nanotubes (CNT) [9]. This can be explained by the fact that ruthenium possesses the optimum nitrogen binding energy to allow both ammonia adsorption and recombinative desorption of nitrogen molecules (the rate limiting step) [10]. Pioneering work in our group has started moving towards the 150–180 °C target by modifying the Ru/CNT catalytic system. For example, the addition of basic promoters such as cesium and graphitisation of the CNT support have led to improvements of the low temperature Ru activity [8, 11].

Doping of CNTs with B, P, F, S and N has been explored in the literature [12]. Within this context, electron rich atoms can offer an enhancement on the electronic properties of the catalytic support. Incorporating these atoms into the CNT structure is thus expected to have comparable benefits to its graphitisation, in an additional effort to enhance the low temperature activity of ruthenium for the release of hydrogen from ammonia.

In this work, we have studied the ammonia decomposition activity of a series of N-doped CNT supports impregnated with 7 wt.% ruthenium and characterised the catalysts with transmission electron microscopy (TEM), temperature programmed reduction (TPR) and CO chemisorption. Herein, we report that the activity of Ru can be enhanced by nitrogen doping of the CNT support due to the increase in support conductivity. However, the synthetic procedure followed for the CNTs nitrogen doping has a critical effect on the reactivity of the final catalyst.

## Experimental

### Nitrogen Doping of CNT Support

Commercial multi-walled CNT were doped with nitrogen by hydrothermal treatment at 180 °C for 12 h with an aqueous ammonia solution (NH_4_OH) of different concentrations. Nitrogen doped CNT prepared with 5 and 15 mL of NH_4_OH are hereafter referred to as N-CNT1 and N-CNT2. Another series of supports were synthesised under analogous conditions but with the addition of 1 g of cetyltrimethylammonium bromide (CTAB). These N-CNT are thus denoted as N-CNT1-CTAB and N-CNT2-CTAB. After treatment, the materials were washed five times with deionised water and dried overnight at 60 °C. Note that unmodified CNT used for comparison are referred to as CNT. The elemental composition and synthesis conditions of all CNT supports used herein are summarised in Table 1.

### Catalyst Preparation and Characterisation

All catalysts were synthesised by incipient wetness impregnation of the different CNT-based materials. For each of the supports, the pore volume was experimentally measured. The corresponding volume of aqueous Ru(NO)(NO_3_)_3_ solution was then added dropwise to each of the supports to achieve a 7 wt.% Ru metal loading. The resulting catalysts were then dried under vacuum at 80 °C for 3 h.

The N:C ratio on CNTs was calculated using high-resolution X-ray photoelectron spectroscopy (XPS) with a Kratos Analytical AXIS-Hsi instrument. The same instrument was also used to determine the nature of the nitrogen species in the CNT supports. TPR and CO pulse chemisorption were carried out using a Micromeritics AutoChem II with a thermal conductivity detector. TPR analyses were performed with 30 mL min^−1^ of 5% H_2_/Ar with a ramp rate of 10 °C min^−1^. The catalysts were degassed at 500 °C for 1 h prior to reduction to remove any residual water or impurities. CO chemisorption of the catalysts was carried out under cryogenic conditions to estimate the metallic surface area and calculate average particle size [13]. TEM of the reduced catalysts was performed using a JEM-200 CX instrument with 200 kV accelerating voltage. Catalysts were prepared for TEM examination by dispersing the catalyst in ethanol and adding one drop to the carbon-coated copper grid. The metal  size distribution is calculated using the diameter of over 100 particles analysed from over ten areas of the TEM grid. Thermogravimetric analyses of the CNT supports were carried out using a Perkin Elmer Pyris 1 TGA using approximately 2 mg of sample which is heated to 100 °C to remove any adsorbed gases and subsequently heated to 600 °C at 10 °C min^−1^ under a constant nitrogen flow at 20 mL min^−1^. The organic groups in the samples were characterized by Fourier transform infrared spectroscopy (FTIR) using a Bio-Rad instrument in the range of 400–4000 cm^−1^ with attenuated total reflection (ATR) mode.

### Testing of Catalytic Activity

Ammonia decomposition activity was tested in a catalytic bespoke flow rig using 25 mg of catalyst diluted in 450 mg of silicon carbide in a packed bed inside a quartz U-shaped reactor. Prior to testing the catalysts were pre-reduced in situ at 230 °C for 30 min with 20 N mL min^−1^ of pure H_2_. The furnace temperature was controlled by an external furnace (Carbolite) with a type K thermocouple located directly above the catalyst bed. A mixture of NH_3_ and He (1:2.4 ratio) was flowed continuously through the reactor with a gas hourly space velocity of ${\text{6}}000~{\text{m}}{{\text{L}}_{{\text{N}}{{\text{H}}_{\text{3}}}}}~{{\text{g}}_{{\text{cat}}}}^{ - {\text{1}}}~{{\text{h}}^{ - \,{\text{1}}}}.$ During the catalytic test the furnace temperature was ramped from ambient temperature to 550 °C at a rate of 2.6 °C min^−1^. The composition of the outlet gas stream was analysed online using a gas chromatographer with a Hayesep Q column and a thermal conductivity detector. The mass balance was closed with ±10% error. The activation energy was calculated with an Arrhenius plot at low levels of conversion (5–20 mol%) to assume a constant apparent rate of reaction.

## Results and Discussion

**Fig. 1:**
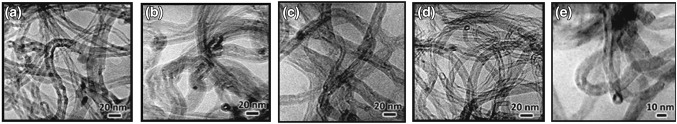
Representative TEM micrographs of CNT supports **a** unmodified CNT, **b** N-CNT1-CTAB, **c** N-CNT2-CTAB, **d** N-CNT1 and **e** N-CNT2

In the first set of experiments, commercial multiwall CNTs were doped with nitrogen using a hydrothermal method in the presence of CTAB surfactant, which is known to enhance the dispersion of CNTs in aqueous media [14]. Despite the fact that N-CNT1-CTAB and N-CNT2-CTAB are synthesised with different amounts of NH_4_OH solution, the resulting N:C ratio is comparable with a nitrogen content of 2.1 and 1.9% respectively (Table 1). This doping method of CNT conserves its morphology as shown in Fig. 1.

**Table 1:** Synthesis conditions and elemental composition of unmodified and N-doped CNTs

Support	C (at%)	N (at%)	N:C ratio	Mass of CTAB in synthesis (g)
CNT	97.3	0.4	0.0041	N/A
N-CNT-CTAB1	96.7	2.1	0.0217	1
N-CNT-CTAB2	96.8	1.9	0.0196	1
N-CNT1	97.5	1.2	0.0123	0
N-CNT2	96.2	1.7	0.0177	0

The catalysts presented herein were all synthesised with 7 wt.% of Ruthenium to address our research hypothesis. Our previous work [11] demonstrated that changing the amount of ruthenium loading (studied in the range from 3 to 13 wt.% Ru) changes linearly only the concentration of active sites without affecting the nature and  type of active site. In this study, we have fixed the ruthenium loading (7 wt.%) for illustration purposes.

**Fig. 2:**
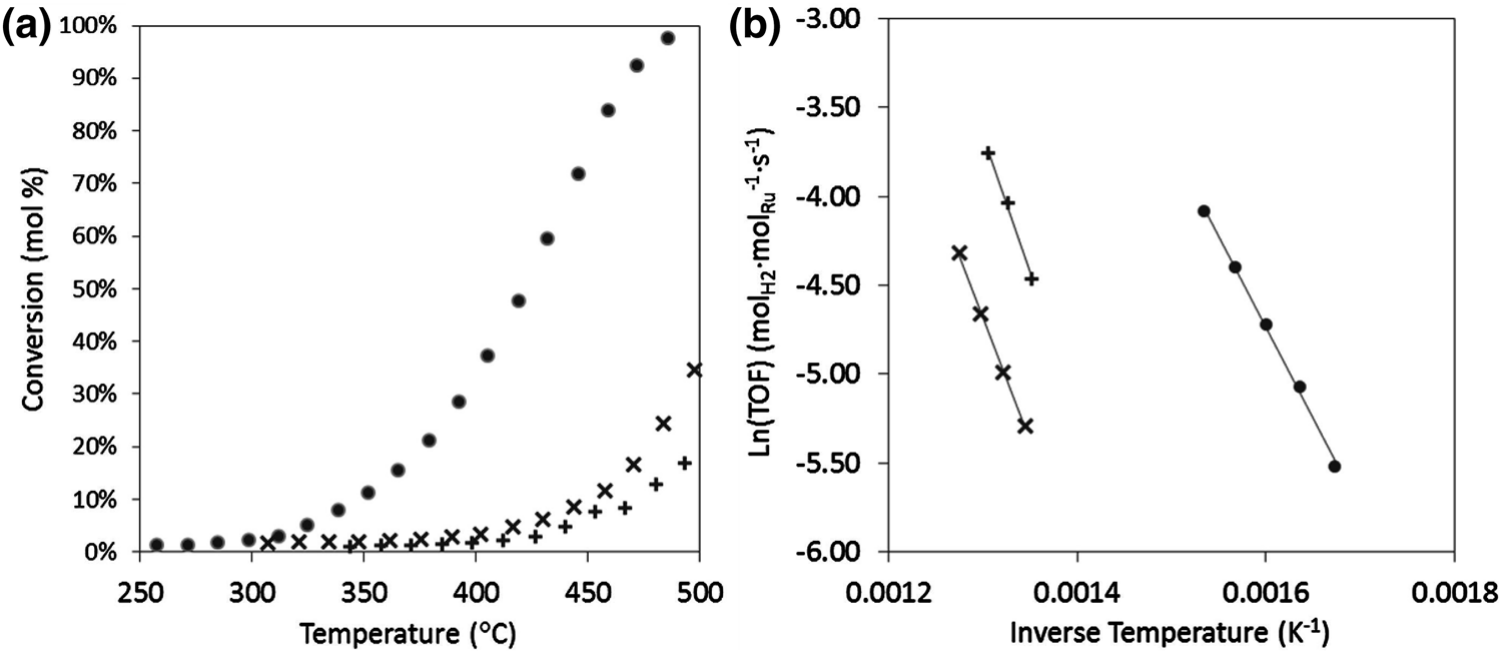
Ammonia decomposition (GHSV = 6000 cm^3^ g^−1^ h^−1^, pre-reduction temperature = 230 °C) **a** conversion versus temperature and **b** Arrhenius plot for 7 wt.% Ru catalysts supported on CNT (*filled circle*), N-CNT1-CTAB (x) and N-CNT2-CTAB (+)

As shown in Fig. 2a, when Ru is added to the N-CNT-CTAB supports, their ammonia decomposition catalytic activity, for a given temperature, significantly decreases compared with the use of the ruthenium supported on unmodified CNT. In addition, the Ru/unmodified CNT catalyst shows hydrogen production from ammonia at temperatures above 320 °C while the Ru/N-CNT-CTAB catalysts only show catalytic activity at temperatures above 430 °C. Interestingly, the activation energy of the nitrogen doped N-CNT-CTAB catalysts increases with respect to the Ru/unmodified CNTS from 81 to 104 kJ mol^−1^ which suggests a modification of the ruthenium active sites.

**Fig. 3:**
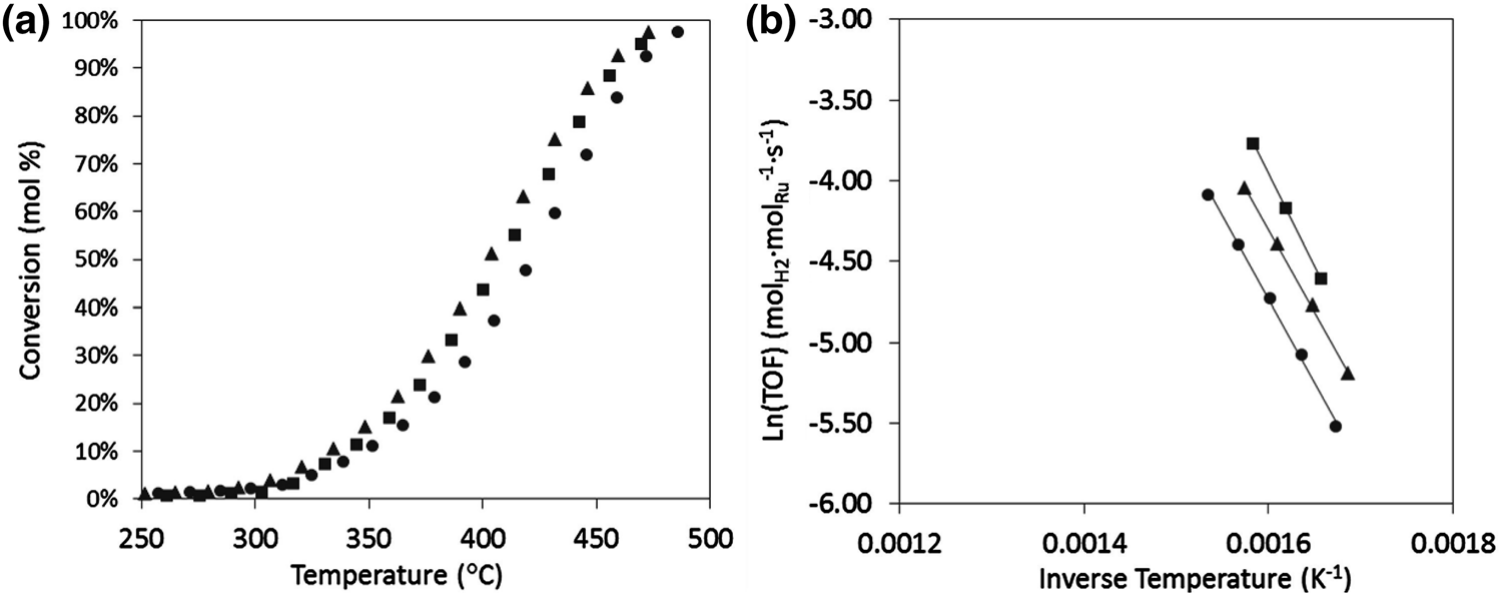
Ammonia decomposition activity (GHSV = 6000 cm^3^ g^−1^ h^−1^, pre-reduction temperature = 230 °C) **a** conversion versus temperature and **b** Arrhenius plot for 7 wt.% Ru catalysts supported on CNT (*filled circle*), N-CNT1 (*filled triangle*) and N-CNT2 (*filled square*)

An alternative method of doping the commercial CNT was developed in the absence of CTAB surfactant. This route results in a slightly lower nitrogen content (1.2–1.7 N at%) compared to the counterpart treatment in the presence of CTAB (Table 1). The second batch of nitrogen modified CNT (N-CNT) were also used to support ruthenium nanoparticles using the same impregnation method as above. In this case, a remarkably different effect is observed on the catalytic activity. Rather than a deterioration in Ru activity, nitrogen doped CNT supports enhance the low temperature ammonia decomposition activity compared to the unmodified CNT as shown in Fig. 3a. However, in all cases (N-doped and unmodified CNTs) show activity from the same temperatures (>300 °C). In addition, the Arrhenius plot in Fig. 3b, shows a similar gradient corresponding to comparable activation energy of the catalysts (~80–85 kJ mol^−1^) which suggest the presence of similar ruthenium active sites in all cases. An increase in Ru activity by N-doping of the CNT support has been previously reported for the ammonia synthesis reaction, which is interesting because the optimal ammonia decomposition catalyst is not necessarily the best for ammonia synthesis due to the difference in conditions and rate limiting steps [15, 16].

One should note in Table 2 the significant difference in turnover frequency (TOF) at 400 °C for the catalysts supported with the N-CNT1-CTAB $({\text{18 mo}}{{\text{l}}_{{{\text{H}}_{\text{2}}}}}~{\text{mo}}{{\text{l}}_{{\text{Ru}}}}^{ - {\text{1}}}~{{\text{h}}^{ - {\text{1}}}}),$ rendering the catalyst almost inactive whereas the use of N-CNT1 support increases the activity compared to the unmodified CNT in terms of TOF from 183 to ${\text{26}}0~{\text{mo}}{{\text{l}}_{{{\text{H}}_{\text{2}}}}}~{\text{mo}}{{\text{l}}_{{\text{Ru}}}}^{ - {\text{1}}}~{{\text{h}}^{ - {\text{1}}}}.$ Scattered studies in the literature use nitrogen doped CNT to enhance the ammonia decomposition activity of Ru nanoparticles, however the studies are focused on higher temperature conditions (> 370 °C), showing lower activity at 400 °C than we report herein or no data at this temperature [17–19]. For example, a Ru–N–C hybrid catalyst using carbon black as the support demonstrated an improvement of high temperature ammonia decomposition activity with the addition of nitrogen but is inactive at 400 °C [19]. We want to highlight the need to move towards lower temperature studies in order to develop viable catalytic systems for ammonia decomposition that can be integrated with a PEMFC.

**Table 2:** Ru properties and catalytic activity of 7 wt.% Ru catalysts on CNT supports

Catalyst	Average Ru particle size (nm)		Ru reduction temperature (°C)	NH_3_ conversion @ 400 °C (mol%)	TOF @ 400 °C $({{\text{L}}_{{{\text{H}}_{\text{2}}}}}\,{\text{mo}}{{\text{l}}_{{\text{Ru}}}}^{ - 1}\,{{\text{h}}^{ - {\text{1}}}})$	TOF @ 400 °C $({\text{mo}}{{\text{l}}_{{{\text{H}}_{\text{2}}}}}\,{\text{mo}}{{\text{l}}_{{\text{Ru}}}}^{ - 1}\,{{\text{h}}^{ - {\text{1}}}})$	E_a_ (kJ mol^−1^)
	TEM	CO chemisorption					
7 wt.% Ru/CNT	2.6 ± 1.3	5.1	150	33.9	4432.7	183	81.4
7 wt.% Ru/N-CNT1	2.4 ± 1.1	7.6	158	48.0	6276.3	260	85.3
7 wt.% Ru/N-CNT1-CTAB	1.9 ± 0.6	4.0	98	3.3	431.5	18	104.1

**Fig. 4:**
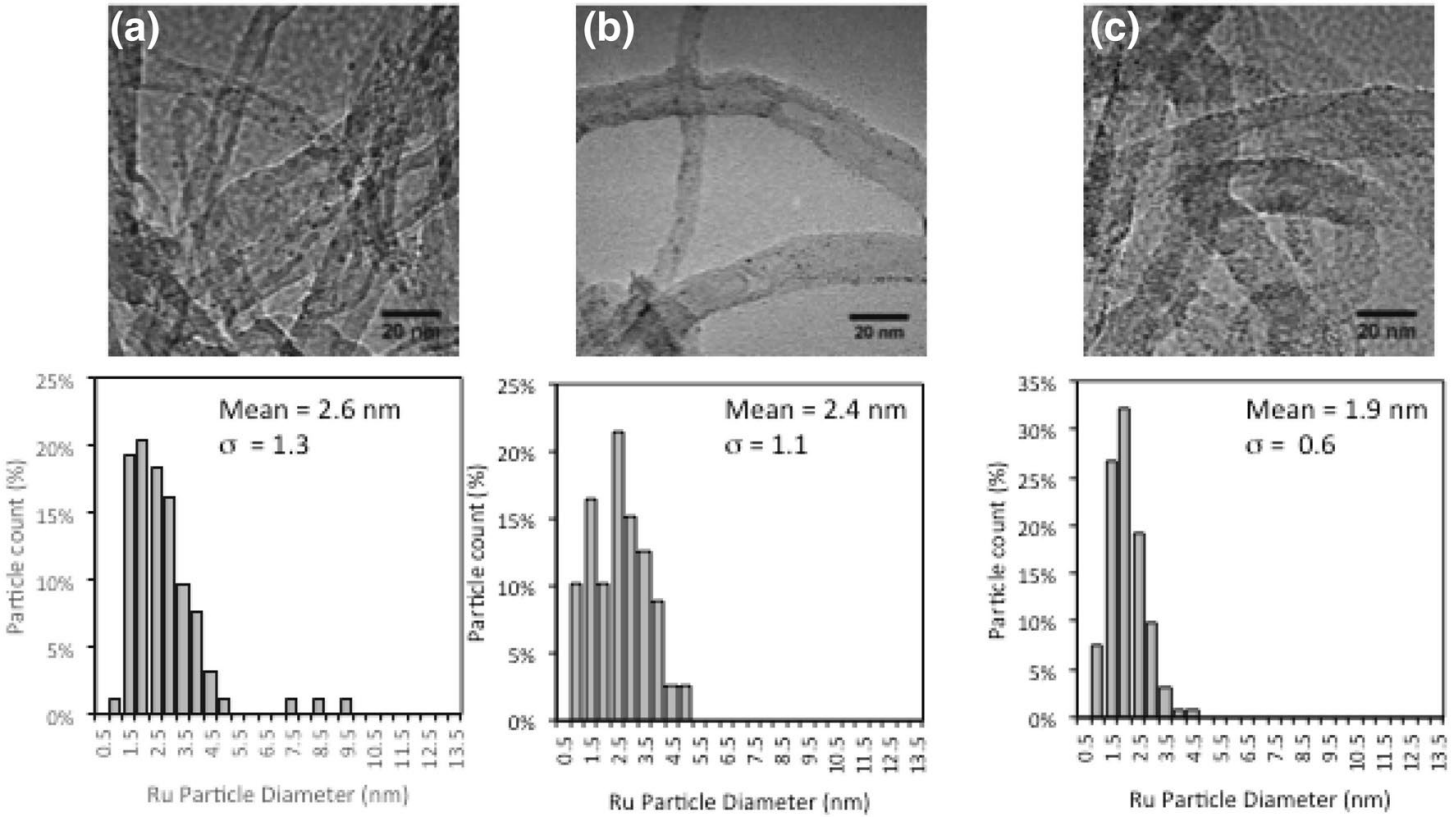
TEM micrographs and Ru particle diameter distributions for reduced catalyst **a** 7Ru/CNT, **b** 7Ru/N-CNT1 and **c** 7Ru/N-CNT1-CTAB

Representative TEM micrographs in Fig. 4 and their corresponding Ru particle size distribution for the reduced catalysts show similar average particle sizes, independent of the addition of nitrogen or the doping preparation method (in the presence or absence of CTAB). This particle size characterisation method estimates ruthenium particles sizes with average values in the range of 1.9–2.6 nm, which are comparable given the standard deviation.

In all cases, a narrow particle size distribution is achieved with standard deviation of the Ru particle size distribution no higher than 1.3 nm. Experimental and theoretical studies have shown that defects arising in the N-CNT structure can act as nucleation points for metal nanoparticle growth, resulting in smaller particle sizes and improved stabilisation [12]. Additionally, empty d-orbitals on Ru^3+^ are able to accept the lone pair of electrons from sp^2^ nitrogen acting as an anchor points against agglomeration [17]. TEM images before and after reduction show similar particle sizes, suggesting negligible effect of sintering.

**Fig. 5:**
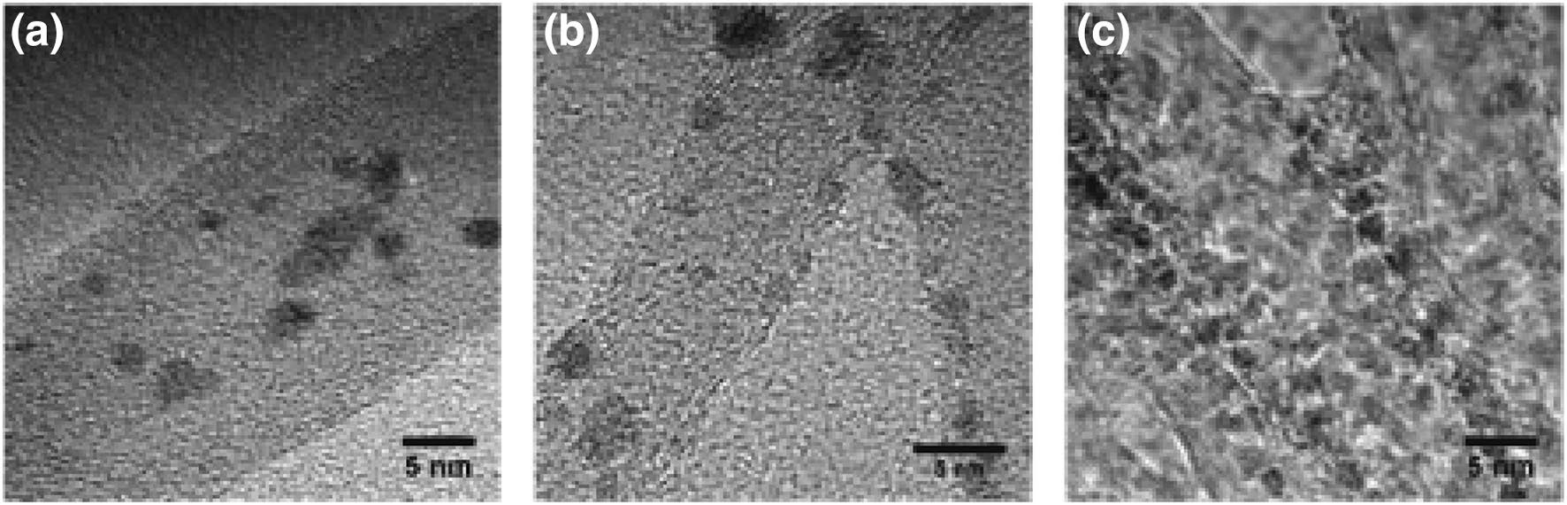
High resolution TEM micrographs for **a** 7Ru/CNT, **b** 7Ru/N-CNT1 and **c** 7Ru/N-CNT1-CTAB

One of the limitations of imaging ruthenium particles is their re-oxidation in contact with air which can potentially underestimate its size. To complement this data, CO chemisorption was carried out after in situ reduction of the catalysts at 230 °C (same conditions as for ammonia decomposition reaction) under cryogenic conditions to avoid the interaction between CO and nitrogen in the pyridinic moieties in the N-CNT. The calculated ruthenium average particle sizes are slightly higher in this case compared to the ones calculated by TEM (Table 2), ranging from 4 to 7.6 nm. However, it is also worth highlighting the possible errors associated with the calculation of metal particles sizes using chemisorption due to the assumption of constant stoichiometric factors, which are known to vary with CO and the assumption of hemispherical particles, which is incorrect based on Fig. 5 showing some axially elongated hemispheres [13].

Computational and experimental studies of Ru clusters suggest that 3–5 nm is the optimal particle size for ammonia decomposition as it maximises the concentration of B_5_ sites, which are believed to be the most active sites for this reaction [20]. Interestingly, the most active catalyst in this study, Ru/N-CNT1, presents a ruthenium particle size outside of this optimum range (based on both TEM and CO chemisorption estimates), suggesting that the electronic modification of the support by the presence of nitrogen plays a highly significant role in enhancing the low temperature ammonia decomposition activity.

**Fig. 6:**
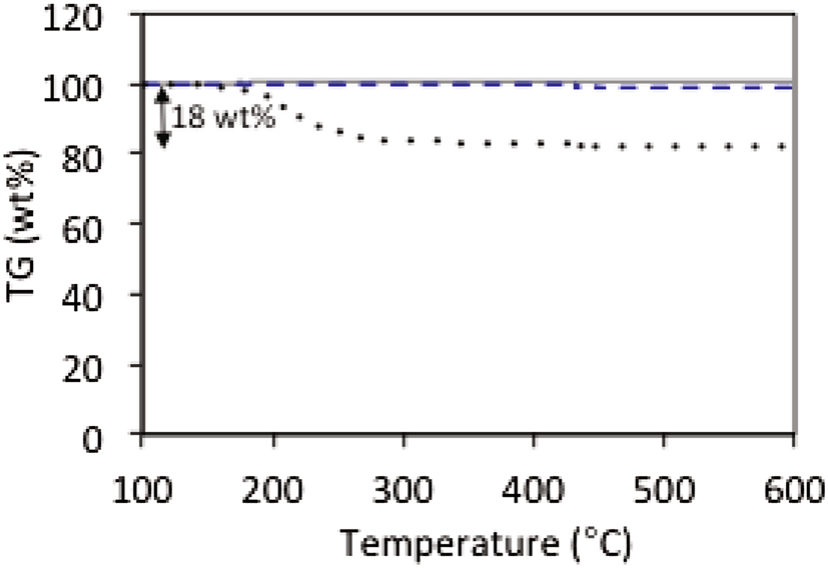
Thermogravimetric analysis of CNT supports: unmodified CNT (*solid grey line*), N-CNT1 (*dashed blue line*) and N-CNT1-CTAB (*black dots*)

TEM micrographs in Figs. 4 and 5 also show that the surface of N-CNT1-CTAB is much well less defined than N-CNT1 and CNT, likely due to the presence of CTAB on the surface. It is important to note that the N-CNT-1-CTAB support did not seem to change its morphology in comparison with the unmodified CNT during the hydrothermal treatment as shown in Fig. 1. However, it seems that the presence of CTAB plays a role during the impregnation method which will consequently affect the ruthenium–support interaction and thus its catalytic activity. In addition, the CTAB molecule contains bromide which might also have a detrimental role in the activity. The residual presence of CTAB can be seen by the TGA plot shown in Fig. 6, in which the unmodified and N-CNT1 supports undergo negligible change in mass up to 600 °C whereas N-CNT1-CTAB experiences a 12 wt.% loss between 200 and 300 °C and an overall 18 wt.% loss during the temperature range studied. Note that all samples are dried in situ for 30 min at 100 °C prior to starting the analysis. Irrespective of the loss of CTAB in N-CNT1-CTAB after 300 °C, the activity of the analogous Ru catalyst does not start until 400 °C (Fig. 2) suggesting that a further increase in temperature is needed to activate Ru once the CTAB has been removed.

**Fig. 7:**
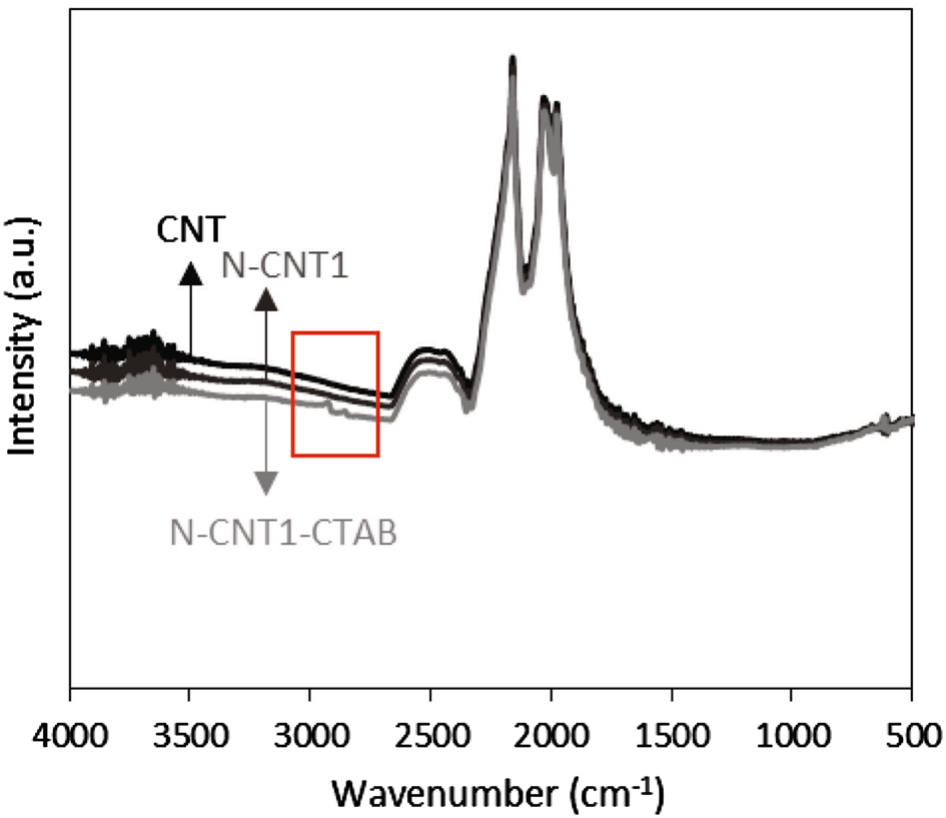
FTIR spectra of CNT supports: unmodified CNT (*solid black line*), N-CNT1 (*solid dark grey line*) and N-CNT1-CTAB (*solid pale grey line*)

The FTIR spectra of unmodified CNT and the nitrogen doped CNT samples are quite similar as shown in Fig. 7. However, the spectrum of the N-CNT1-CTAB support shows two spectra show two FTIR bands centred at 2925 and 2854 cm^−1^ (surrounded by the red box in Fig. 7) which can be assigned to the C–H stretching mode of the methyl (–CH_3_) group in aliphatic compounds, present in the structure of CTAB. This result provides further evidence of the presence of CTAB in N-CNT1-CTAB, offering an explanation to the difference in catalytic performance reported. No information about nitrogen species in the CNT supports is obtained from the FTIR spectra.

**Fig. 8:**
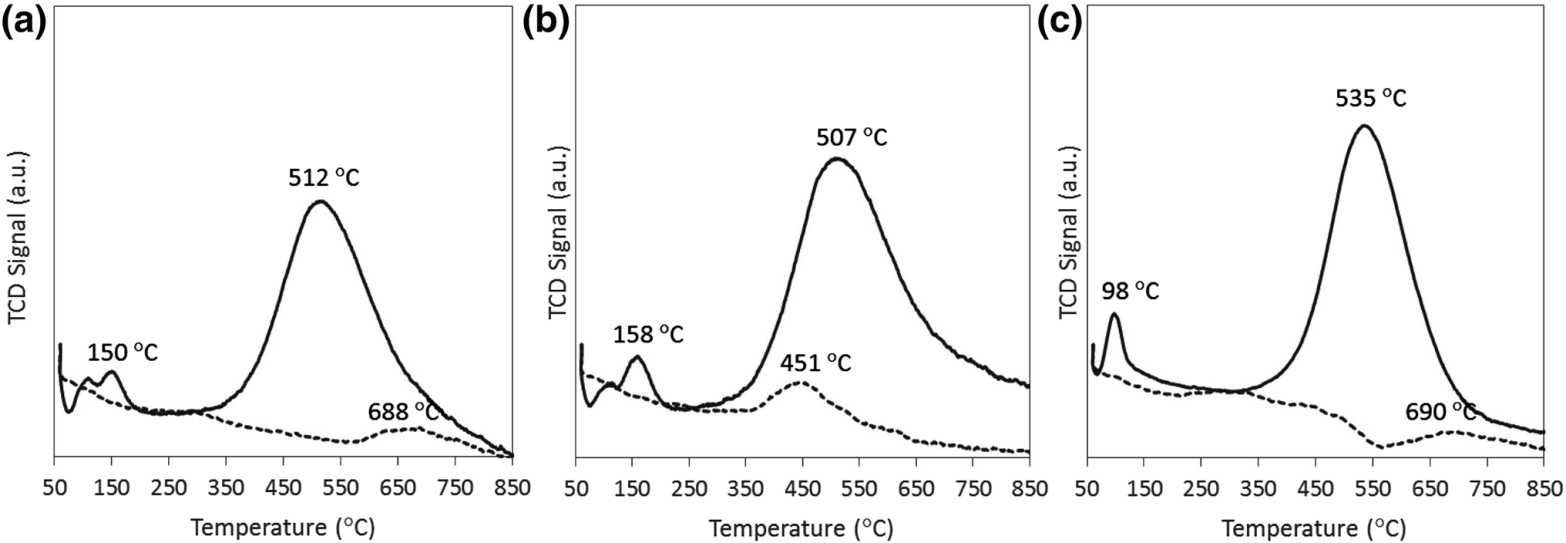
Temperature programmed reduction profile for catalysts supported on **a** CNT, **b** N-CNT1 and **c** N-CNT1-CTAB in which support only is shown by *dashed line* and support with 7 wt.% Ru catalyst is represented by a *solid line*

TPR of the different catalysts also provide insights about the metal–support interaction. As shown in Fig. 8, the reduction temperature of Ru slightly increases to 158 °C from 150 °C when supported on N-CNT compared to CNT, suggesting a small variation in the strength of the metal–support interaction from nitrogen doping of the support. This observation is in agreement with the electronic modification of the ruthenium active site and consequently the enhancement of its catalytic activity for ammonia decomposition at low temperatures. By contrast, the temperature of the reduction peak for ruthenium supported on N-CNT-CTAB is significantly lower at ~100 °C (Fig. 5c), indicative of a weak metal–support interaction which in contrast to the previous case, has a detrimental effect on the resulting catalytic activity. The relatively small size of the reduction peak at 98 °C (Fig. 8c) suggests that some Ru may already be reduced from the drying process during synthesis, likely due to the poor metal–support interaction.

**Fig. 9:**
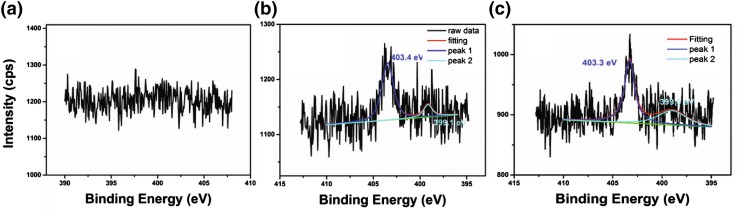
XPS spectra in the N 1 s region for **a** CNT, **b** N-CNT1 and **c** N-CNT1-CTAB

Nitrogen doping of CNT results in pyridinic rings within the rolled graphitic sheets of CNTs as carbon atoms are replaced with electron rich nitrogen atoms [21]. When the nitrogen atom in the ring is located at the edge of the sheet, they are referred to as pyridinic; when the nitrogen atom is placed within the structure, they are referred as graphitic or quaternary [12]. Both of these species are detected by the XPS spectra of the supports shown in Fig. 9, identifying two peaks in the N 1 s region for the N-CNT1 and N-CNT1-CTAB support. The peak centred around 403.3 eV can be assigned to quaternary nitrogen and the other peak centred at 399.0 eV is attributed to pyridinic nitrogen [18]. The peaks are only observed for the N-doped CNT and not in the unmodified CNT (Fig. 9a). Depending on the doping conditions, the formation of oxides is also possible as well as five-membered rings containing nitrogen called pyrroles [12]. However, NOx and pyrrolic species exhibit a peaks at 404.9–405.6 and 399.8–400.8 eV respectively, which are not observed by XPS for the nitrogen doped CNT reported herein (Fig. 9) but previously identified in other nitrogen-doped CNTs materials [18] which suggests that the doping method has an effect on the nature of the nitrogen species present on the CNTs.

The higher electronegativity of nitrogen with respect to carbon and its lone pair of non-bonding electrons make it an electron rich source able to interact with ruthenium nanoparticles. Thus, nitrogen doping of the CNT support enhances the catalytic activity by electronically modifying the ruthenium active sites as shown in Fig. 3. In agreement with our results, N-CNT has also been reported to increase the catalytic activity of nickel nanoparticles, with the distinct electronic properties attributed to activation of the Ni d-orbitals by nitrogen [22].

The replacement of carbon atoms with nitrogen atoms also results in an increase in support basicity. Doping CNT with nitrogen is thus analogous to adding a basic promoter such as cesium, which are known to enhance the Ru/CNT catalytic activity in the ammonia decomposition reaction by promoting the Ru^δ−^ sites and consequently promoting the associative desorption of nitrogen which is the rate limiting step at low temperatures [11]. In a similar manner, ruthenium active sites can be electronically modified by increasing the CNT conductivity by graphitisation [8, 23]. The increase in graphitisation of CNT by nitrogen doping has been reported in the literature, evidenced by a graphitic carbon peak observed at 26.4° with powder X-ray diffraction and a graphitic band at 1553–1601 cm^−1^ in Raman spectroscopy [21]. Thus, our results herein demonstrate that nitrogen doping of CNT is an alternative way of modifying the conductivity and basicity of the support and subsequently enhancing the catalytic activity of ruthenium for the ammonia decomposition reactions at low temperatures.

It is likely that higher nitrogen doping of the CNT would result in further enhancement of the ammonia decomposition activity, however, the doping levels that can be achieved using the hydrothermal method cannot exceed the nitrogen content levels of 1–2 at% reported here [24]. It may be possible to increase the nitrogen content by carrying out the doping in the presence of an oxygen source, such as acetonitrile or ethylbenzoate [21]. However, the N-CNT produced in this way are reportedly less graphitic in nature, which is likely to be detrimental to the ability of the support to electronically modify Ru [21]. Chen et al. [17] used a microwave plasma method to dope CNT with nitrogen and obtained similar nitrogen loadings as we present here, however their catalyst showed little ammonia decomposition activity at 400 °C. In any case, it is difficult to predict the effect of compounds added during synthesis on the final activity, as we have shown that doping with CTAB has a detrimental effect on the final catalyst.

Indeed, despite the clear effect of the addition of nitrogen into the CNTs structure on the resulting activity of the ruthenium-based catalyst, additional aspects need to be considered to elucidate the promotion mechanism. Small differences in N:C ratio between N-CNT1 and N-CNT2 does not seem to significantly affect the activity as shown in Fig. 2, although in both cases the activity is higher than the counterpart ruthenium catalyst supported on the unmodified CNT. Similarly, the difference in nitrogen content on the equivalent ruthenium catalysts doped in the presence of CTAB does not significantly affect the activity (Fig. 3). The final N:C ratio is slightly higher in the latter case (N-CNT-CTAB) likely due to the enhanced dispersion of the CNTs in the presence of CTAB during doping as mentioned above and also due to the potential presence of CTAB (C_19_H_42_BrN) traces in the catalysts.

## Conclusions

Our results herein demonstrate that the replacement of carbon atoms with nitrogen in CNTs supports facilitates electronic modification of ruthenium due to the improved conductivity of the support leading to higher TOF in the ammonia decomposition reaction compared to the counterpart catalysts supported on unmodified commercial CNT. Furthermore, nitrogen doping increases the basicity of CNT and thus provides similar benefits to the addition of a basic promoter reported elsewhere. Thus, nitrogen doping simultaneously combines the benefits of graphitisation of CNT and the addition of a basic promoter. The use of CTAB in the nitrogen doping process results in a low activity catalyst as the presence of CTAB weakens the ruthenium–support interaction and consequently its electronic modification.
